# The role of intravascular imaging in chronic total occlusion percutaneous coronary intervention

**DOI:** 10.3389/fcvm.2023.1199067

**Published:** 2023-09-11

**Authors:** Iosif Xenogiannis, Antonis N. Pavlidis, Thomas E. Kaier, Angelos G. Rigopoulos, Grigoris V. Karamasis, Andreas S. Triantafyllis, Panos Vardas, Emmanouil S. Brilakis, Andreas S. Kalogeropoulos

**Affiliations:** ^1^ Department of Cardiology, Mitera General Hospital, Hygeia HealthCare Group, Athens, Greece; ^2^ Second Department of Cardiology, Attikon University Hospital, National and Kapodistrian University of Athens Medical School, Athens, Greece; ^3^ Department of Cardiology, St Thomas’ Hospital, Guy’s and St Thomas’ NHS Foundation Trust, London, United Kingdom; ^4^ Department of Cardiology, Asklepion General Hospital, Athens, Greece; ^5^ Center for Coronary Artery Disease, Minneapolis Heart Institute and Minneapolis Heart Institute Foundation, Minneapolis, MN, United States

**Keywords:** chronic total occlusion (CTO), intravascular imaging, intravascular ultrasound (IVUS), optical coherence tomography (OCT), CTO crossing, stent optimization

## Abstract

Chronic total occlusions (CTOs) represent the most complex subset of coronary artery disease and therefore careful planning of CTO percutaneous coronary recanalization (PCI) strategy is of paramount importance aiming to achieve procedural success, and improve patient's safety and post CTO PCI outcomes. Intravascular imaging has an essential role in facilitating CTO PCΙ. First, intravascular ultrasound (IVUS), due to its higher penetration depth compared to optical coherence tomography (OCT), and the additional capacity of real-time imaging without need for contrast injection is considered the preferred imaging modality for CTO PCI. Secondly, IVUS can be used to resolve proximal cap ambiguity, facilitate wire re-entry when dissection and re-entry strategies are applied and most importantly to guide stent deployment and optimization post implantation. The role of OCT during CTO PCI is currently limited to stent sizing and optimization, however, due to its high spatial resolution, OCT is ideal for detecting stent edge dissections and strut malapposition. In this review, we describe the use of intravascular imaging for lesion crossing, plaque characterization and wire tracking, extra- or intra-plaque, and stent sizing and optimization during CTO PCI and summarize the findings of the major studies in this field.

## 1. Introduction

**Figure 1:**
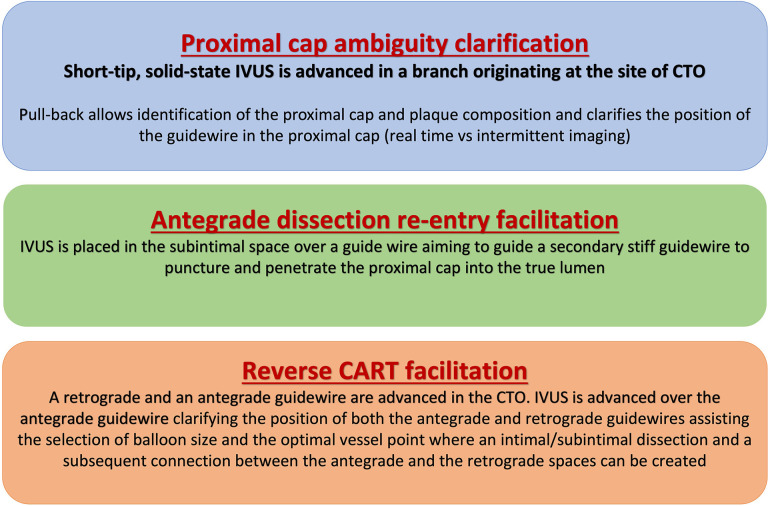
Applications of intravascular ultrasound for facilitating chronic total occlusion crossing. CART, controlled antegrade and retrograde tracking; CTO, chronic total occlusion; IVUS, intravascular ultrasound.

Chronic total occlusion (CTO) percutaneous coronary intervention (PCI) can be challenging. Over the last years the refinement of the CTO PCI techniques, the evolution of CTO crossing algorithms as well as the substantial advancements in available equipment have resulted in substantial improvement of CTO PCI success rates (85%–90% in experienced centers with a dedicated CTO PCI program). However, CTO PCI outcomes are still considered inferior compared to PCI of non-occlusive lesions (1). Multiple factors encompassing the underlying pathophysiology of CTOs, such as the extensive atherosclerotic disease, heavy calcification especially in patients with previous coronary artery bypass surgery (CABG), negative vessel remodeling distal to the CTO segment that can potentially lead to the implantation of undersized stents can increase the likelihood of stent failure, thus compromising the long-term outcomes after CTO PCI (2–4). Intravascular imaging modalities, namely intravascular ultrasound (IVUS) and optical coherence tomography (OCT) can help to overcome several challenges associated with CTO PCI, i.e., wire crossing and guide stent optimization, potentially improving both the procedural success rates and the long-term clinical outcomes (5). In this review we describe the most common intravascular imaging techniques that are used to facilitate CTO PCI (Figure 1) and we summarize the findings of the major studies on the field.

### 1.1. Technical characteristics, utilization recommendations and penetration of intravascular imaging modalities

IVUS and OCT are currently the two commercially available intravascular imaging modalities for PCI. IVUS image acquisition is based on the production of ultrasound waves, whereas OCT utilizes near infrared light for imaging. Image acquisition requires the advancement of an imaging probe, over a 0.014″ guidewire, into the coronary artery of interest, followed by an automated or manual (only for IVUS) pullback. There are two main types of IVUS catheters, the first one involves the solid-state electronic phased array IVUS transducers such as the Eagle Eye (Koninklijke, Philips N.V., Amsterdam, Netherlands) and the second one involves the mechanically rotating transducers, such as the OptiCross HD (Boston Scientific, Natick, Massachusetts) and Refinity (Koninklijke, Philips N.V., Amsterdam, Netherlands) (6). IVUS has a greater penetration depth compared with OCT, whereas OCT has a better spatial resolution. The major differences between the technical features of these modalities are outlined in Table 1. Due to its greater penetration depth, the capacity of manual pullback and steady probe positioning in specific areas of interest that might require continuous real time visualization such as in ambiguous proximal caps to facilitate proximal cap identification and wire penetration, IVUS is currently considered the most used imaging modality for CTO PCI. Furthermore, OCT requires antegrade contrast injections to clear the column of blood from the coronary artery and allow the light beam to reach the vessel wall for successful image acquisition. This can cause further hydraulic dissection of the vessel wall and expansion of the subintimal haematoma impeding the wire re-entry process. Moreover, the extra dye contrast can increase the overall administered contrast volume and thus increase the risk of contrast-induced acute kidney injury. The latter is especially important in the setting of CTO PCI during which a large volume of contrast is usually required (7, 8). Nevertheless, given its higher spatial resolution, OCT is highly valuable for assessing distal edge dissections or stent strut malapposition (9).

**Table 1:** Comparison between intravascular ultrasound and optical coherence tomography technical characteristics and applications in chronic total occlusion interventions.

	IVUS	OCT
Source of image	Ultrasound (wavelength 40 µm)	Near infrared light (wavelength 1.3 µm)
Axial resolution	100–150 µm	10–20 µm
Transverse resolution	200 µm	20 µm
Penetration depth	8 mm	2 mm
Acquisition speed	0.5–1 mm/s	25 mm/s
Applications in CTOs	crossing, stent optimization	stent optimization
Disadvantages:	cannot image through calcium	contrast injection needed, limited penetration

CTOs, chronic total occlusions; IVUS, intravascular sound; OCT, optical coherence tomography.

Numerous CTO wire crossing algorithms have been developed over the last decade (10–15). The majority of these algorithms recommends the use of IVUS to resolve proximal cap ambiguity and facilitate antegrade proximal cap puncture. The Asian Pacific and Japan CTO club algorithms also endorse the use of IVUS for re-entering into the distal true lumen in cases of antegrade wiring, when the guidewire has entered into the extraplaque space using the parallel wire technique (12, 13). Currently the use of intravascular imaging is considered a fundamental tool during CTO PCI for selecting the appropriate stent size, perform adequate plaque characterization (calcium identification and quantification) and finally facilitate the subsequent stent optimization after stent implantation (16).

There is a variable penetration of intravascular imaging worldwide, mainly due to differences in availability, cost issues and operators training and familiarity with the relevant equipment. The frequency of IVUS use in CTO PCI in the western world ranges from 12% to 37% (17–20), whilst it is substantially higher among Korean and Japanese operators (39–47.8%) (21, 22). Use of IVUS for CTO PCI has been increasing over time (19, 20) whereas use of OCT is infrequent (3% in the PROGRESS-CTO registry) (23).

### 1.2. Intravascular imaging for facilitating CTO crossing

#### 1.2.1. Ambiguous proximal cap

Proximal cap ambiguity is defined as the failure to clearly determine the proximal entry point into the CTO segment due to a flush ostial occlusion or the presence of concealing or overlapping branches that cannot be resolved despite multiple angiographic projections (24, 25). Proximal cap ambiguity is encountered in approximately 30% of CTO lesions and is associated with lower procedural success and more frequent attempts for retrograde crossing (24, 26). Computed tomography angiography can help to delineate vessel course and proximal cap position before the procedure, whilst during the procedure, IVUS with real-time fluoroscopy can effectively resolve proximal cap ambiguity (27, 28).

**Figure 2:**
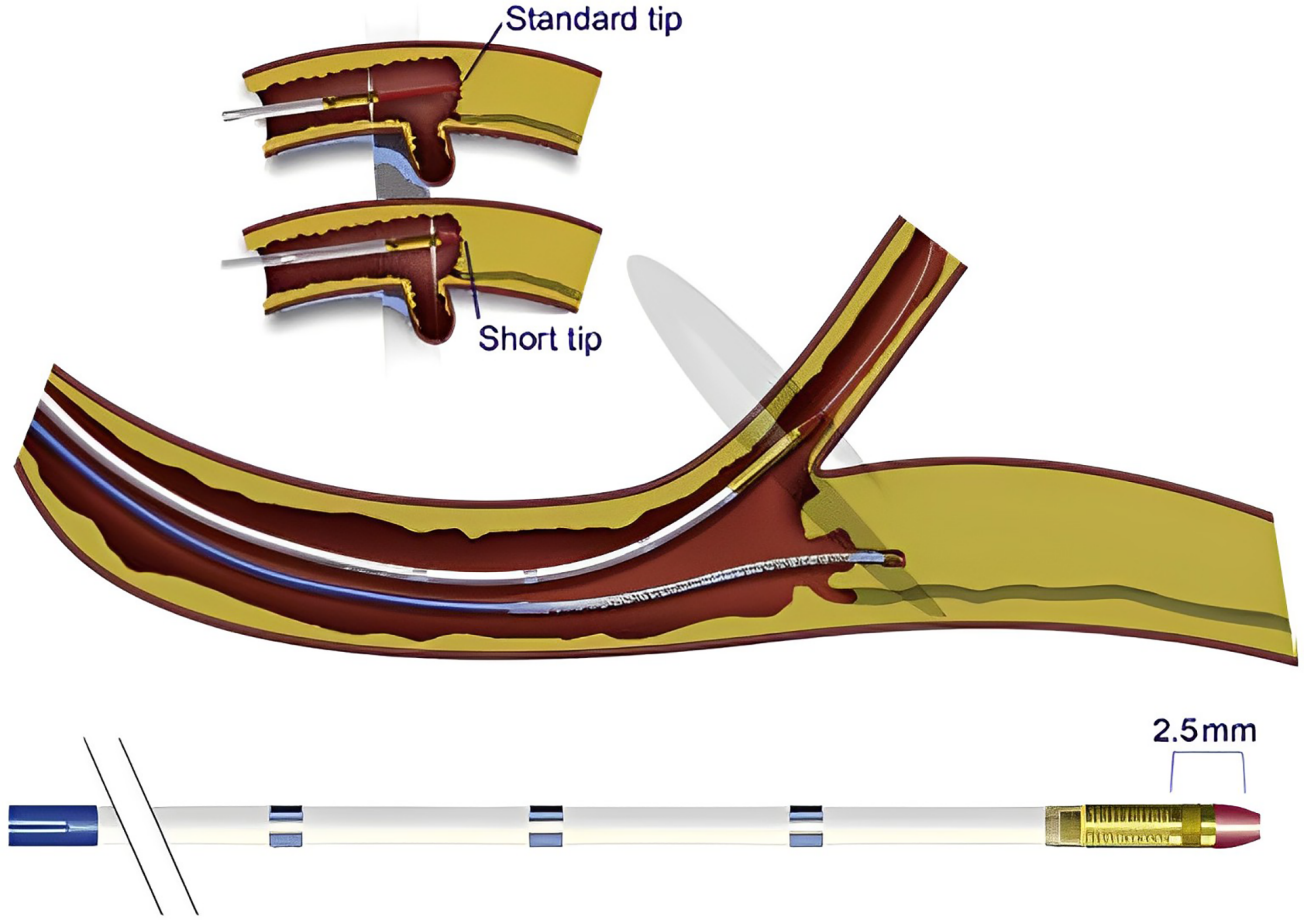
Short tip IVUS catheter entering side branch to guide CTO crossing. With permission from Brilakis ES. Manual of chronic total occlusion interventions a step-by-step approach. Second edition. ed. London: Elsevier/Academic Press; 2018.

A short-tip, solid-state IVUS catheter such as the Eagle Eye (Koninklijke, Philips N.V., Amsterdam, Netherlands) is preferred in CTO PCI, as it is more deliverable and can image close to its tip, contrary to the rotational IVUS catheters that usually have a 2–3 cm distal monorail segment, that requires more distal advancement of the catheter tip (Figure 2). On the other hand, smaller profile rotational IVUS catheters can be more advantageous as they can be inserted in smaller side branches (29).

**Figure 3:**
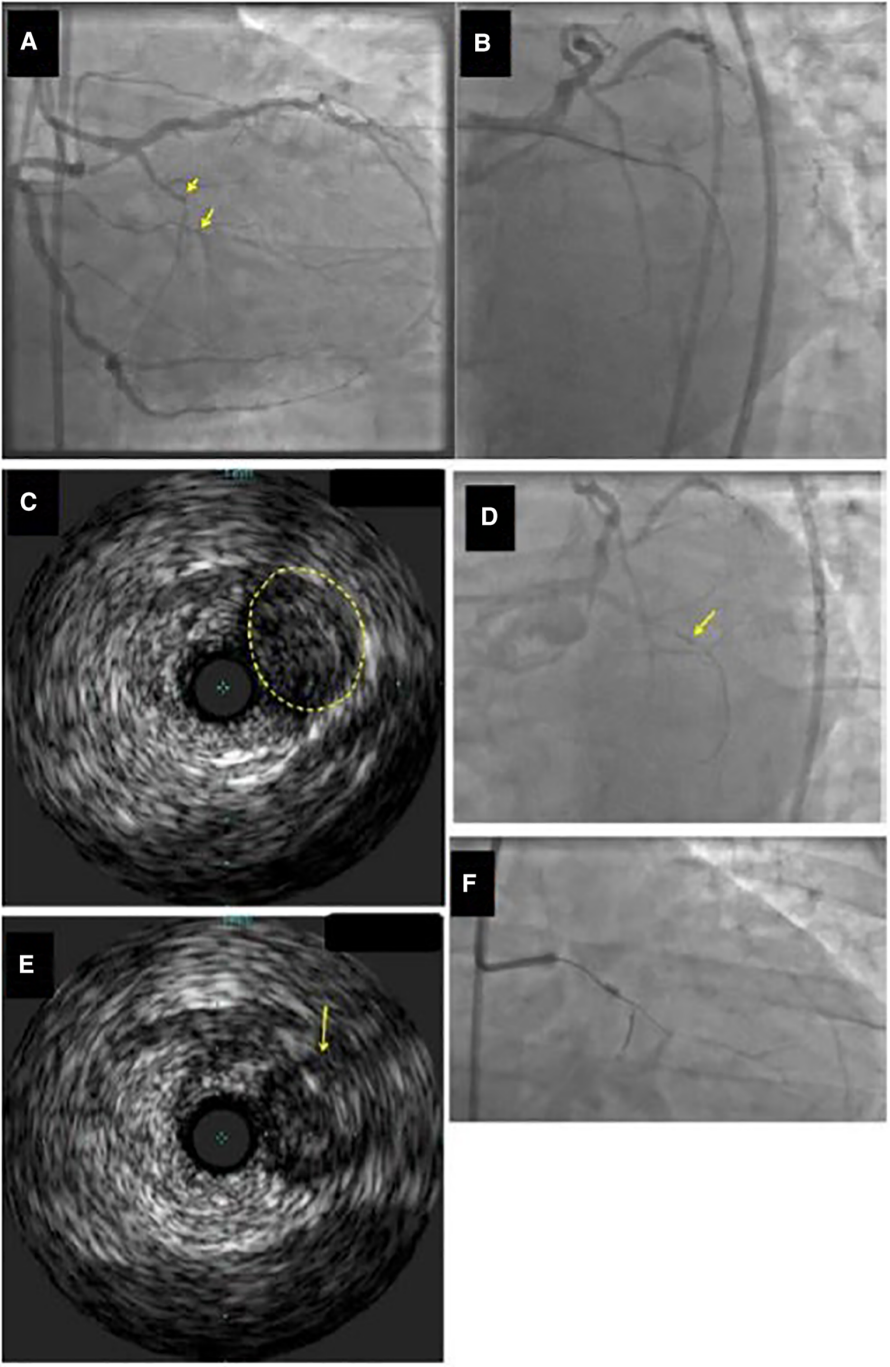
Example of identification of the proximal cap location by intravascular ultrasound (IVUS). (**Panel A**) Ostial chronic total occlusion (CTO) of the first obtuse marginal branch (arrow). (**Panel B**) The guidewire kept entering the distal circumflex during antegrade wire escalation approach. (**Panel C,D**) IVUS demonstrated that the CTO (yellow circle in **Panel C**) originated proximal (arrow in Panel D) to the distal circumflex's apparent origin. (**Panel E**) A Confianza Pro 12 guidewire was used for antegrade crossing and its location within the CTO was confirmed by IVUS. (**Panel F**) Confianza Pro 12 was advanced through the occlusion. Modified with permission from Brilakis ES. Manual of chronic total occlusion interventions a step-by-step approach. Second edition. ed. London: Elsevier/Academic Press; 2018.

The IVUS catheter is advanced over the guidewire into a side branch that originates at the site of the vessel occlusion next to the proximal cap. At this stage careful evaluation of the side branch is essential to ensure that the side branch is large enough to accommodate the IVUS catheter and the branch is not extremely tortuous and calcified to allow the smooth insertion and advancement of the catheter. The subsequent pullback allows identification of the proximal cap in most of the cases (Figure 3) unless there is severe calcification (30). Alternatively, in cases of ostial branch occlusion, IVUS insertion in the main vessel can reveal the proximal point of the occlusion (29). Two different techniques have been previously described: (a) simultaneous real-time imaging during crossing attempts, or (b) intermittent (serial) imaging with IVUS catheter withdrawal to allow wire crossing attempts, followed by re-insertion of IVUS to define the exact guidewire position.

In a small size guiding system such as a 6Fr guiding catheter, simultaneous use of an IVUS catheter and a microcatheter for real-time imaging to facilitate proximal cap wire crossing is not feasible and therefore intermittent imaging can be used instead. On the other hand, with larger guiding catheters such as 7 or 8Fr, simultaneous use of an IVUS catheter and a microcatheter with a guiding wire is possible, allowing real-time intravascular imaging during wire-crossing attempts. In practical terms, the choice between the two techniques usually depends on various factors including operator preference, complexity of the lesion and available resources.

Intravascular imaging with IVUS will help to define the composition of the plaque (calcified or fibrous) and help to choose between a more penetrating guide wire and a polymer jacketed wire and after crossing to confirm the position of the guidewire within the proximal cap and inside the vessel structure. If the latter is confirmed, then a microcatheter can be advanced over the wire to further facilitate crossing. Otherwise, the wire is withdrawn and repositioned (29). In a study of 22 patients with ostial, stumpless CTOs, IVUS guided PCI resulted in 77% procedural success (31). IVUS carries the risk of thrombosis and side branch injury, especially in small and tortuous side-branches.

#### 1.2.2. Antegrade re-entry

During antegrade crossing the guidewire can enter the extraplaque space leaving the operator 4 options: (a) re-entering into the distal true lumen preferably using a dedicated re-entry system such as the Stingray balloon catheter (Boston Scientific) or Recross dual lumen microcatheter; (b) redirecting the guidewire; (c) using the parallel wire technique; or (d) trying to cross to the distal true lumen with a second guidewire under IVUS guidance.

Regarding IVUS-assisted wire crossing, a stiff guidewire is selected for the advancement of IVUS in the extraplaque space. Predilatation with a low profile (1.0–1.5 mm) balloon can facilitate IVUS advancement. With this technique, a 7 or 8 French guide catheter is used in order to accommodate the IVUS catheter, whilst a second stiff guidewire [such as Gaia 3rd, Confianza Pro 12 (Akatsuki-Cho, Seto-Shi, Aichi, Japan) or Hornet 14 (Boston Scientific, Natick, Massachusetts)] and a microcatheter are used to re-enter into the distal true lumen (29).

The previously described technique is among the most difficult techniques during CTO PCI and is mostly used by experienced Japanese CTO operators (12, 13).

More recently, a new IVUS based technique, the IVUS-3D wiring has been described to facilitate wire-based re-entry, when the antegrade wire has been inadvertently tracked inside the subintimal space (32). With this technique a specific CTO IVUS device the AnteOwl IVUS (AO-IVUS) (Terumo, Tokyo, Japan) is being used to detect the exact position of the tip of a second stiffer guide wire, which will be used for re-entry into the distal true lumen. As the integrated IVUS pull-back system allows the CTO operator to perform multiple pullbacks, the operator can precisely detect the shaft and the tip of the second guidewire, without increasing the risk of subintimal space and haematoma expansion. Using the combination of IVUS and orthogonal fluoroscopic projections the operator can define the exact position of the tip of the wire and direct the wire puncture into the distal true lumen. The additional favorable CTO features of the AO-IVUS catheter CTO dedicated system include a short tip to transducer distance of only 8 mm to minimize vessel trauma and expansion of the subintimal haematoma, a long monorail distance to facilitate IVUS catheter deliverability and finally a small shaft profile of only 3.1 Fr to allow simultaneous use with a microcatheter and a second guidewire in a 7Fr guiding catheter (32).

#### 1.2.3. Reverse controlled antegrade and retrograde tracking (*reverse CART*)

Retrograde crossing has significantly increased the success of CTO PCI over the last two decades from 70% to 90% (33). Indications to use a retrograde approach involve proximal cap ambiguity, bifurcation at the distal cap, diffusely diseased distal vessel and flush aorto-ostial occlusion (34). CTO lesions where retrograde crossing is attempted are often more complex and have higher J-CTO scores (35, 36).

**Figure 4:**
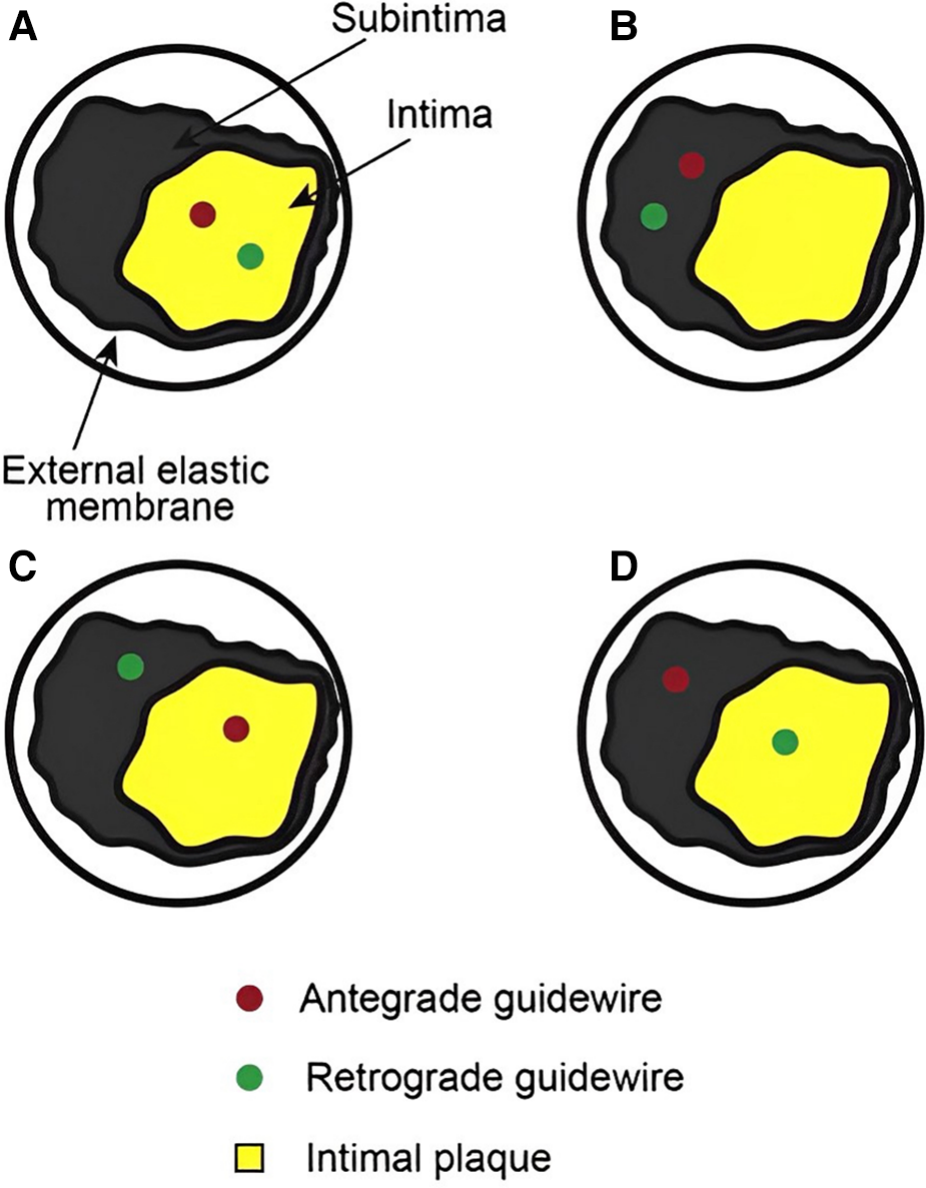
Antegrade and retrograde guidewire positions as assessed by IVUS in reverse CART. (**A**) Antegrade and retrograde guidewires are both within intimal plaque. This is the ideal scenario to make a connection, after antegrade balloon dilation in the chronic total occlusion body. If needed, retrograde puncture of intimal plaque with a stiffer wire could be performed. (**B**) Antegrade and retrograde guidewires are both within the subintimal space. This is another ideal condition in which it is easy to create a connection in the same space after balloon dilation. (**C**) Antegrade guidewire in intimal plaque but retrograde guidewire in subintimal space. This is a very complex situation in which it is crucial to create a medial disruption with proper balloon sizing to create a connection between the two guidewires. In case of failure, it may be possible to advance the antegrade wire distally to enter the subintimal space and create the previous condition (subintimal–subintimal). (**D**) Antegrade wire in subintimal space but retrograde wire in intimal plaque, often very calcified. This is the most complex situation because antegrade balloon dilation usually enlarges the subintimal space (increasing intramural hematoma) with low probability of creating a connection between the two guidewires. In this situation, the connection is usually achieved by pushing the retrograde wire in the subintimal space (usually with retrograde knuckle technique). In such a complex case, a possible less-used alternative is retrograde balloon dilation (original CART) to create medial dissection and facilitate antegrade guidewire connection with the retrograde guidewire. CART, controlled antegrade retrograde tracking; IVUS, intravascular ultrasound. Modified with permission from Galassi AR, Sumitsuji S, Boukhris M, et al. Utility of intravascular ultrasound in percutaneous revascularization of chronic total occlusions: an overview. JACC Cardiovasc Interv 2016; 9:1979–91, Elsevier*.* Used with permission from Brilakis ES. Manual of chronic total occlusion interventions a step-by-step approach. Second edition. ed. London: Elsevier/Academic Press; 2018.

Reverse CART is considered the contemporary retrograde crossing technique of choice during CTO PCI (35). An antegrade wire, usually in a knuckle configuration, is advanced into the CTO segment, followed by the inflation of a balloon (1:1 size) creating a localized extraplaque dissection and establish a connection between the antegrade and the retrograde space. A retrograde guidewire is then advanced in a similar fashion from the distal true lumen to the newly created extraplaque space and then into the proximal true lumen (37). However, reverse CART has inherent limitations that should always be taken into account: first the technique cannot provide any information regarding the size of the vessel, secondly the frequently encountered uncertainty regarding the true position (intraplaque vs. extraplaque) of both guidewires (Figure 4) can hamper the effort to make a successful connection between the antegrade and retrograde spaces and thus impede retrograde wire crossing into the proximal true lumen. IVUS-guidance can help to overcome these limitations (38). The IVUS can reliably define the position of the antegrade and retrograde guidewires (intraplaque vs. extraplaque), measure the vessel size within the CTO segment, characterize plaque composition at the overlapping segment of both guidewires (soft, dense fibrous, calcific), and finally assist with the selection of the optimal balloon size and the most favorable segment of the vessel where an intraplaque/ extraplaque dissection and a subsequent space connection can be created (37).

**Figure 5:**
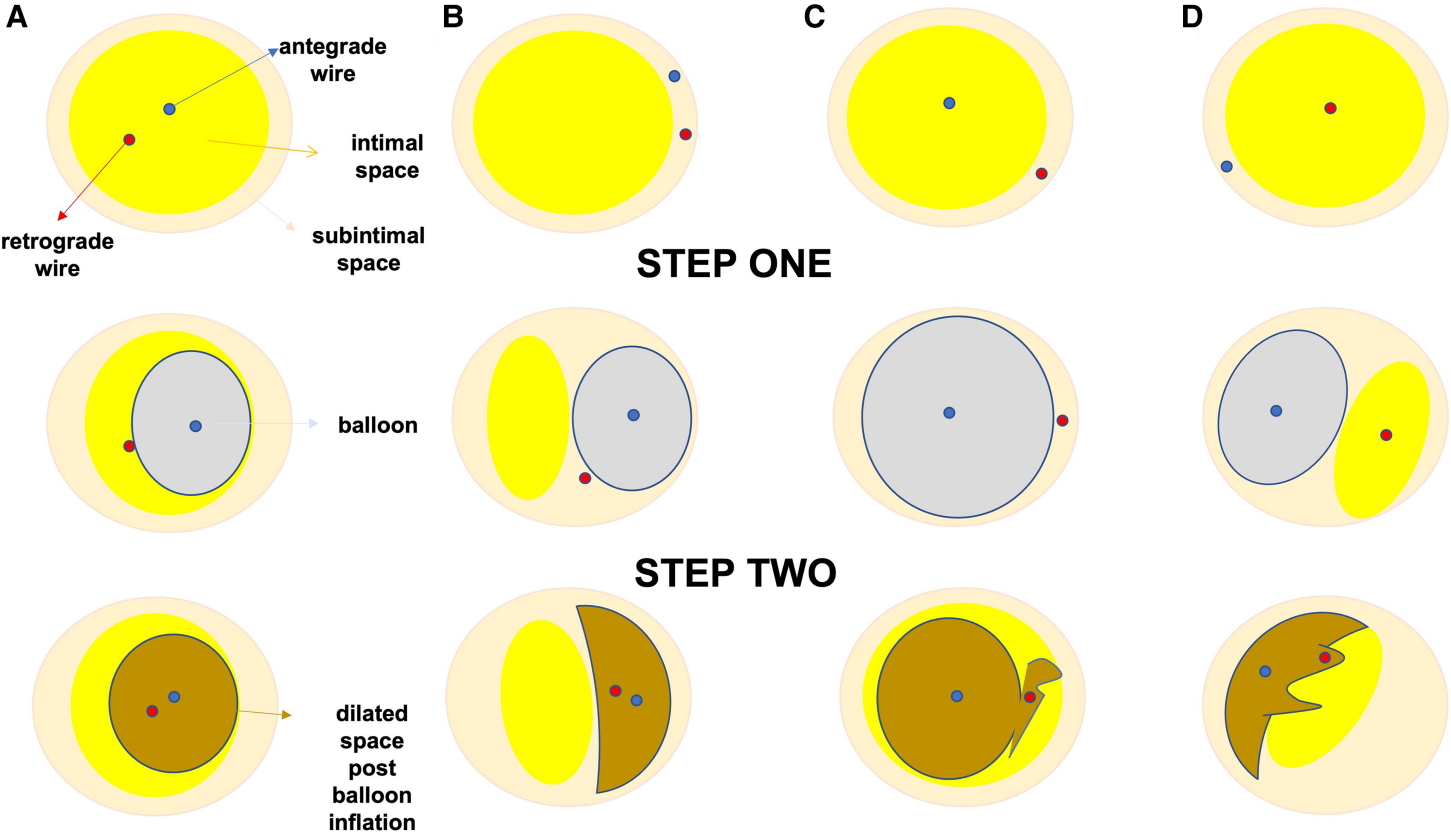
Proposed algorithm for IVUS use to facilitate reverse CART during CTO PCI. (**A–D**) corresponds to the various scenarios during reverse—CART. In (**A**) both antegrade and retrograde guide wires are positioned in the intraplaque space, in (**B**) both guide wires are in the subintimal space, in (**C**) the antegrade wire is intraplaque, whilst the retrograde wire is subintimal and finally in (**D**) the antegrade wire is subintimal, whilst the retrograde wire is intraplaque. During step 1, IVUS catheter is advanced over the antegrade wire in the overlapping segment between the antegrade and the retrograde wire. After the appropriate positioning of the IVUS catheter we aim to determine the true position of the two guide wires (intraplaque or subintimal). In step 2, according to each scenario we proceed with the most suitable strategy modification. For scenario A, we choose an 1 to 1 sized balloon to dilate the antegrade space and then with the support of a guide-extension catheter (Teleflex Trapliner is recommended as the integrated trapping balloon will facilitate exchange equipment) we aim to advance the retrograde wire inside the antegrade guide extension catheter. In scenario (**B**), where both guide wires are still in the same space but subintimally, we follow the same approach. In scenario (**C**), where the antegrade wire is intraplaque and the retrograde wire is subintimal we opt to dilate the antegrade space with larger balloons in order to create a connection-fenestration between the antegrade and the retrograde space and facilitate the retrograde wire, usually a stiff hydrophilic guide wire with good torqueability such as a Gaia 3rd (ASAHI INTECC), advancement into the antegrade space and guide catheter extension. In scenario (**D**), where the antegrade wire is subintimal and the retrograde wire is intraplaque, we aim to use extra-stiff retrograde guide wires such as Hornet 14, Confianza pro 12 in order to create a connection and facilitate retrograde wire tracking from the retrograde intraplaque position to the antegrade subintimal space and thus successful reentry into the antegrade space with retrograde wire advancement into the antegrade catheter. In the last two scenarios the additional use of the DRAFT technique (Deflate and Retract of the antegrade balloon followed by immediate advancement of the retrograde stiff guide wire) will further facilitate successful retrograde wire externalization.

The steps of the technique are as follows:

- After crossing the collateral vessels with a guidewire and microcatheter, retrograde CTO segment wiring is performed using a medium weight polymer jacketed guidewire [(ASAHI Gladius MG (Akatsuki-Cho, Seto-Shi, Aichi, Japan) or PILOT 200 (Abbott Vascular, Santa Clara, California)] (38).
- The antegrade guidewire is advanced into the CTO segment. A balloon sized 1:1 is inflated inside the CTO segment preferably avoiding the areas of large plaque accumulation such as the proximal or distal caps, aiming to create intimal and medial disruption and subsequently to achieve a connection between the antegrade and retrograde spaces.
- The IVUS is advanced over the antegrade guidewire [(preferably a short-tip solid-state IVUS catheter such as Eagle Eye Short Tip (Koninklijke, Philips N.V., Amsterdam, Netherlands)] allowing the detection of the true position of the guidewires (intraplaque vs. extraplaque), the presence of a plausible connection between the two guidewires and finally the estimation of plaque volume and qualitative characteristics (such as excess calcium) (29).
- If there is a connection between the antegrade and the retrograde space (Figure 5A,B), the utilization of gradually larger balloons can facilitate the successful advancement of the retrograde guidewire into the proximal true lumen. In this scenario, the most likely causes of failure to advance the retrograde guidewire into the proximal true lumen are extensive disease and heavy calcification, and extreme tortuosity or recoil proximal to the point of connection (39). Delivery of a guide catheter extension just proximal to where the connection has been made, usually abolishes this problem, allowing retrograde guidewire externalization.
- If there is no connection between the two guidewires and the antegrade wire is intraplaque while the retrograde wire is extraplaque (Figure 5C), it is recommended to use the largest allowed antegrade balloon based on IVUS sizing to facilitate wire connection after balloon inflation. A second option is to use a higher penetration force retrograde guidewire in order to try to puncture into the antegrade space (39). In case of failure, it is suggested to move proximally or distally the site at where reverse CART is attempted. The transit balloon technique and retrograde crossing under direct IVUS visualization are the last resorts. With regards to the transit balloon technique an antegrade balloon is inflated into the antegrade space with a simultaneous attempt to puncture the balloon with a wire. After confirming balloon puncture and retrograde wire position in the antegrade space, the antegrade balloon is deflated and retracted, whilst the retrograde wire is advanced forward (39).
- The most difficult scenario is the absence of connection between the two guidewires when the antegrade wire is extraplaque, but the retrograde wire is intraplaque. In this scenario, larger antegrade balloon dilations are unlikely to create a connection and resolve the problem. IVUS can help to identify this difficult scenario (Figure 5D) and facilitate the connection between the 2 wires. Retrograde crossing with puncturing with a very stiff guidewire such as Gaia 3rd, Confianza Pro 12 (Akatsuki-Cho, Seto-Shi, Aichi, Japan) or Hornet 14 (Boston Scientific) from intraplaque into the antegrade extraplaque space is reasonable and it is encouraged. Crossing can be performed under direct antegrade IVUS guidance. Some operators recommend to inflate a large balloon over the antegrade guidewire during retrograde puncture attempts in order to avoid antegrade extraplaque space collapse (39). If the previous technique fails, a knuckled Pilot 200 (Abbott Vascular, Santa Clara, California) or Gladius MG (Akatsuki-Cho, Seto-Shi, Aichi, Japan) guide wire can be advanced retrogradely increasing the chances for entering the subintimal space and making connection between the two guidewires. If all attempts fail, change to traditional CART or using the confluent balloon technique can be attempted.

The four different clinical scenarios regarding the various positions of the guidewires are illustrated in Figure 4. Figure 5 illustrates a proposed algorithm on how to use IVUS to facilitate wire re-entry during reverse—CART.

In the first landmark analysis of the IVUS-facilitated CTO PCI, successful revascularization was achieved in all 31 patients included in the study. Interestingly 22 of 31 patients (71%) had previous failed CTO recanalization attempts (38). Dai et al. used IVUS-guided reverse CART in 49 patients with prior failed CTO PCI. IVUS guidance was implemented successfully in 96% of the cases aiding the operators to achieve high rates of technical (96%) and procedural (94%) success (40, 41).

### 1.3. Plaque and vascular injury characterization during CTO PCI

#### 1.3.1. Calcified lesions

**Figure 6:**
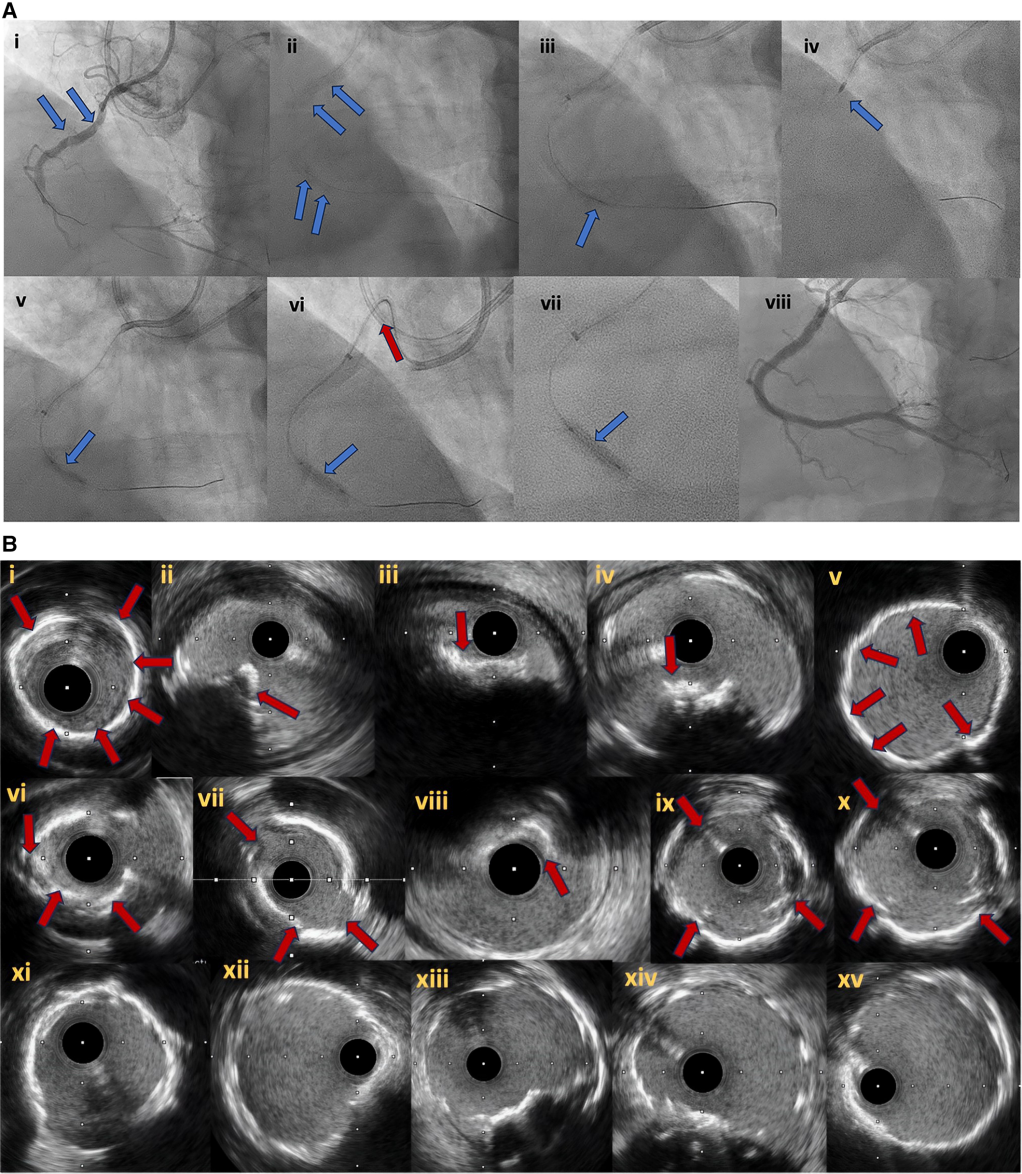
(**A**) Right coronary artery CTO (**6Ai**). In (**6Ai, Aii**) angiographic evidence of heavy calcification is noticed (blue arrows). Grenadoplasty is performed (**6Aiii**, blue arrow) to facilitate microcatheter and balloon crossing followed by rotational atherectomy. Undilatable lesion (**6Av**, blue arrow) was successfully treated with intravascular lithotripsy (**6Avi, Avii**, blue arrows). In (**6Avi**), the tension to the guide extension and catheter during balloon forward motion due to heavy vessel calcification is noticed (red arrow). Final angiographic result after successful CTO recanalization and plaque modification with combined grenadoplasty, rotational atherectomy and intravascular lithotripsy (**6Aviii**). (**B**) intravascular ultrasound pictures. Different modes of calcification including circumferential thick calcification (**6Bi, 6Bv**) and calcified nodules (**6Bii–iv**, red arrows). The effect of plaque modification techniques (grenadoplasty, rotational atherectomy and intravascular lithotripsy) is demonstrated in (**6Bvi–x**). Final intravascular images after successful stents insertion (**6Bxi–xv**).

Percutaneous coronary interventions in CTOs with heavy calcification are associated with worse outcomes, higher rates of in-stent restenosis and target lesion failure (42). In addition, moderate to severe CTO calcification is associated with lower technical and procedural success rates, longer procedural and fluoroscopic times, higher radiation dose, higher contrast dye volume use and almost double rates of major adverse events compared to CTOs with mild or no calcification (43). Therefore, the characterization of calcium distribution and quantification are considered invaluable steps to further guide intraprocedural strategies during CTO PCI (Figures 6A,B).

Intravascular imaging can differentiate between concentric and eccentric distribution of calcium. Eccentric calcification in the form of calcified nodules can increase the risk of partial stent expansion and subsequently worse outcomes after CTO PCI (44). In addition, intravascular imaging in the form of IVUS has shown superior sensitivity compared to plain angiography in identifying calcification during CTO PCI (96% vs. 61%) (45).

Even though, OCT has better capacity to quantify calcium thickness compared to IVUS, the latter is the preferred imaging modality during CTO PCI as the OCT requires vigorous contrast injections that can potentially expand coronary dissection and intramural haematomas.

#### 1.3.2. Subintimal tracking, intramural haematoma and dissections

Although antegrade wire-based crossing techniques are successful in approximately 50% of CTO PCIs, in more complex occlusions advanced dissection and re-entry techniques either antegrade or retrograde are required to achieve successful CTO recanalization (46).

**Figure 7:**
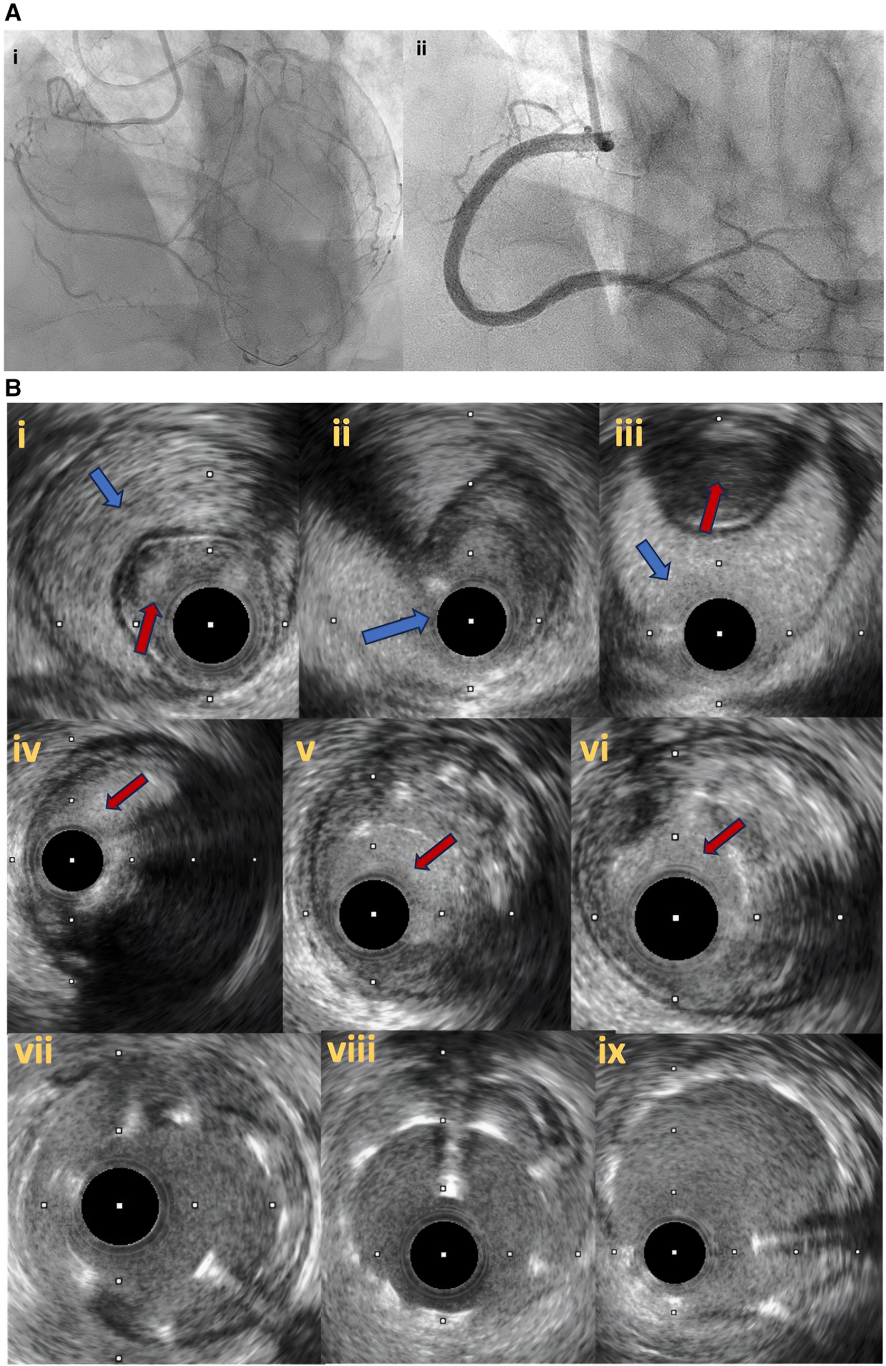
(**A**) Right coronary artery total occlusion revascularization with antegrade dissection reentry technique (**7Ai–ii**). (**B**) Intravascular ultrasound pictures after successful distal true lumen reentry and CTO recanalization. In (**7Bi**), intraluminal wire position (red arrow) with large subintimal haematoma is noticed. In (**7Bii**), the transition from true lumen into subintimal space is noticed (blue arrow), whilst in (**7biii**) the wire is in the subintimal space (blue arrow) that compresses the true lumen (red arrow). In (**7Biv–vi**), intraplaque wire tracking is noticed. Final IVUS images after stent deployement (**7bvii–ix**).

**Figure 8:**
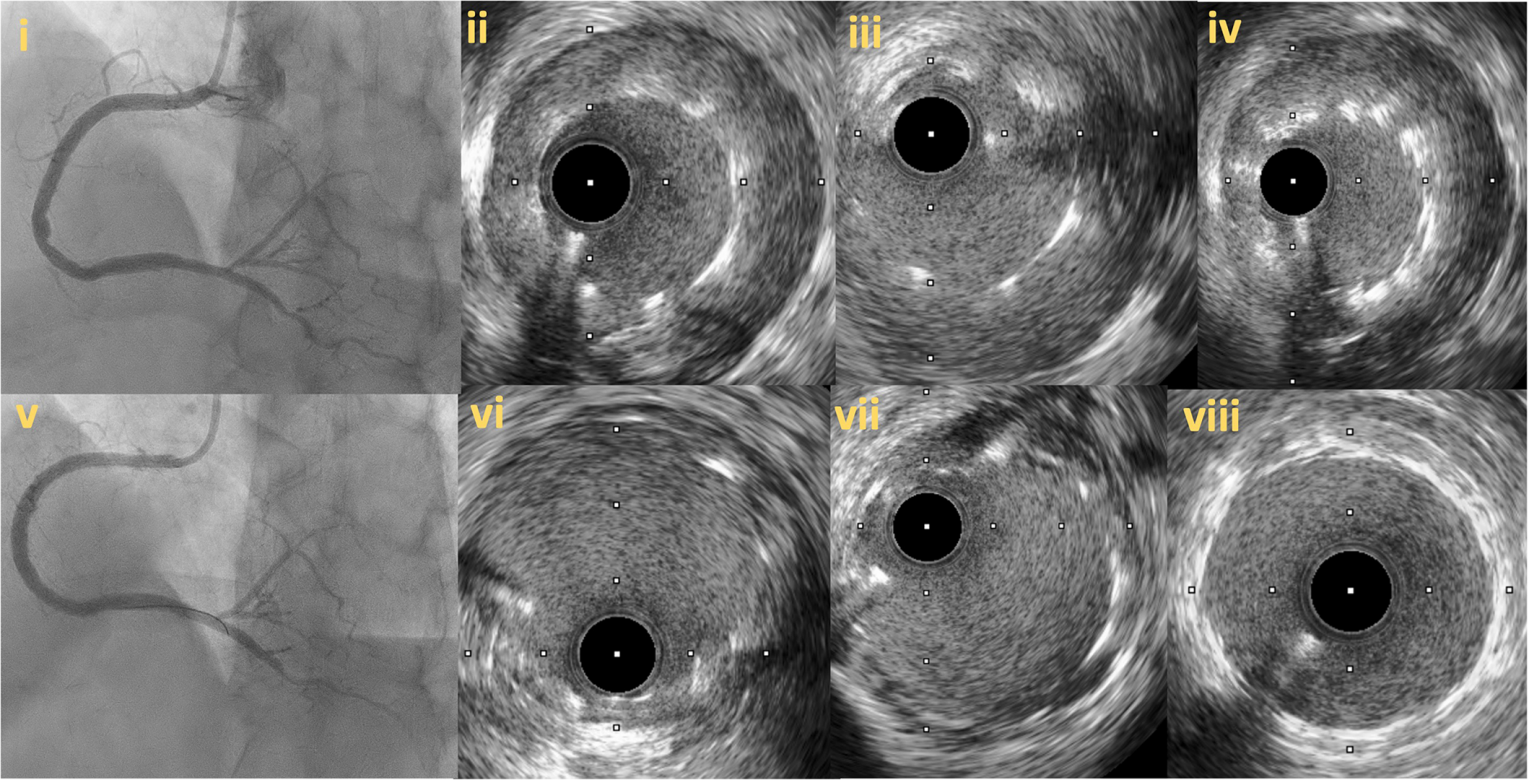
Follow up angiography in patient illustrated in figure 7, 3 months after successful RCA CTO PCI (**8i**). IVUS demonstrates significant stent vessel size mismatch and significant malapposition due to subintimal haematoma healing and vessel positive remodeling after successful PCI (**8ii–iv**). Angiographic (**8v**) and IVUS images (**8vi–viii**) after stent optimization to correct stent-vessel size mismatch and malapposition.

Even though the intended successful crossing technique is believed to correctly reflect the actual pattern of guidewire tracking (wire escalation corresponds to intraplaque course of the wire whilst dissection and re-entry implies some component of subintimal wire tracking 47), two previous studies have demonstrated a significant discordance in approximately 15%–20% of cases between presumable intended crossing technique and actual crossing technique rates as these were identified by IVUS (48, 49). Precise identification of wire tracking, subintimal or intraplaque, could be associated with important clinical implications as subintimal tracking can be associated with larger vessel injury and intramural haematomas and dissection, implantation of longer stents and potentially higher complication rates and adverse events (49). In patient with large haematomas, follow-up angiography in 3-months’ time to allow appropriate vessel healing might be considered to identify late malapposition and vessel stent size mismatch and further optimize stent expansion and apposition, whilst in segments with subintimal wire tracking avoidance of balloon inflation at high pressures is advisable to prevent vessel perforation (Figures 7A,B, 8).

### 1.4. Stent sizing and optimization

**Figure 9:**
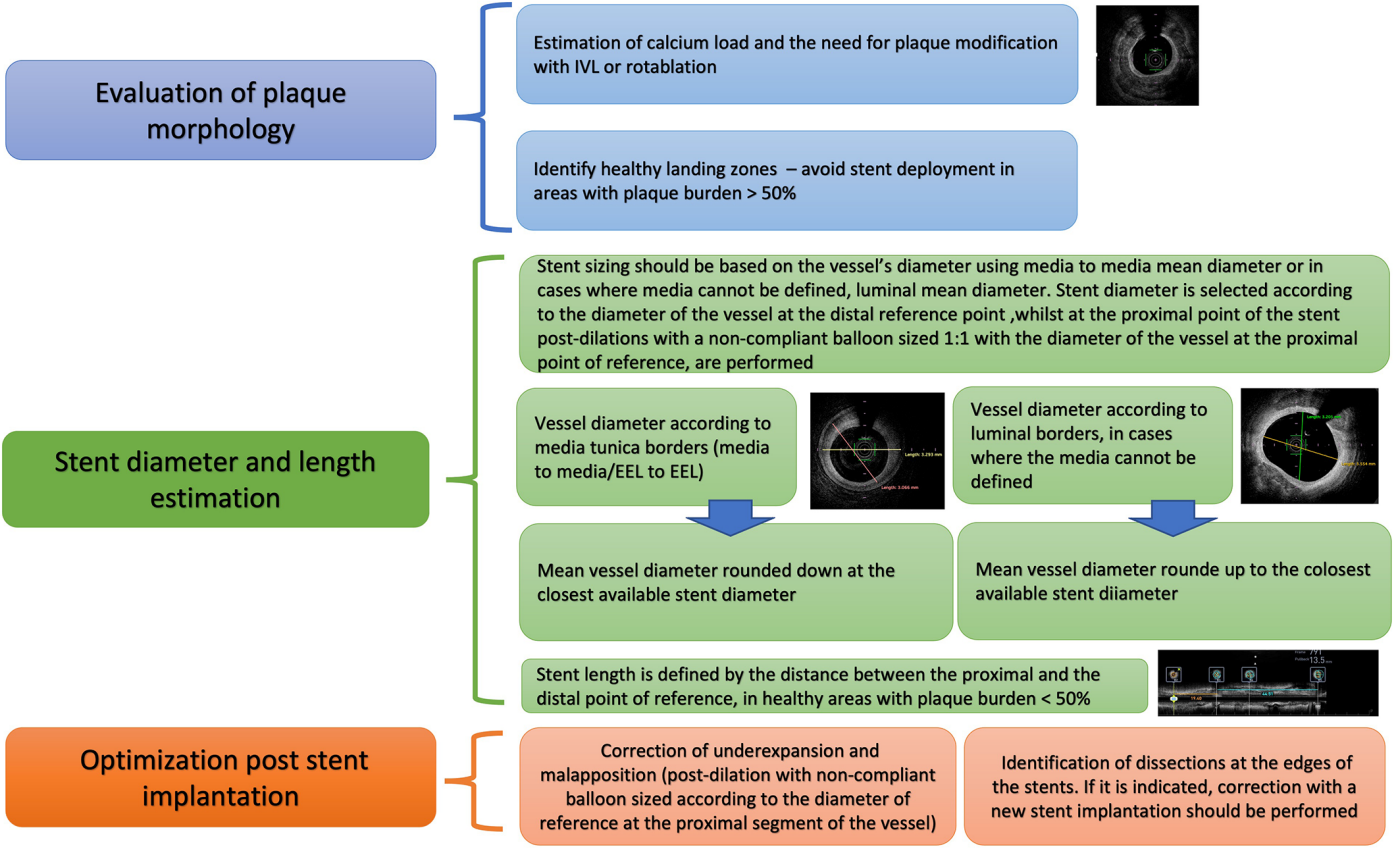
Proposed algorithm for intravascular imaging guided CTO PCI: precise evaluation of plaque characteristics and proper guidance for plaque modification, estimation of stent diameter and length and stent optimization post stent implantation. IVUS, Intravascular ultrasound; CTO, Chronic total occlusions; PCI, Percutaneous Coronary Intervention; EEL, external elastic lamina.

**Figure 10:**
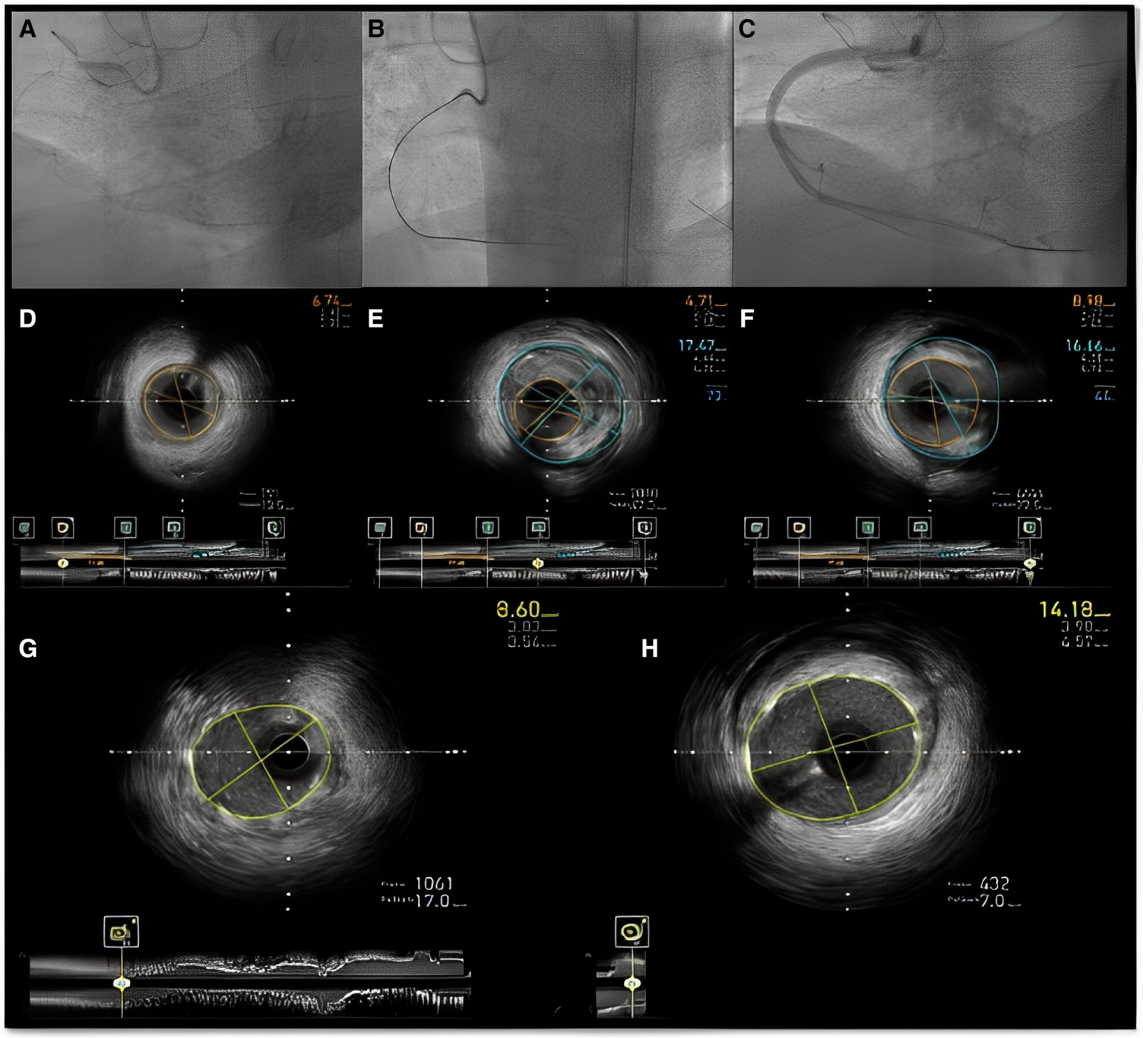
Intravascular imaging (IVUS) guided chronic total occlusion (CTO) percutaneous coronary intervention; (**A**) initial angiography with contralateral injections to define CTO lesion characteristics; (**B**) successful antegrade wire crossing; (**C**) final angiographic result after IVUS guided drug eluting stent (DES) implantation; (**D**) distal landing zone with reference cross sectional area and diameter estimated at 3 mm; (**E**) CTO segment cross-sectional area and reference diameter estimated at 4.5 mm; (**F**) proximal landing zone with reference cross sectional area and diameter estimated at 4.5 mm. The overall lesion length requiring stent coverage from distal landing zone was estimated at 65 mm and therefore two overlapping DES 3.0 × 38 mm and 4.0 × 38 mm were successfully implanted and post-dilated with 3.5 and 4.5 mm non-compliant balloons based on media to media reference diameters as per IVUS measurements; (**G**) distal stent cross-sectional area with excellent absolute (>5.5 mm^2^) and relative expansion (>100%); (**H**) proximal stent cross-sectional area with excellent relative expansion (>100%). Used with permission by Elsevier. Kalogeropoulos AS, Alsanjari, O, Davies, JR. et al. Impact of Intravascular Ultrasound on Chronic Total Occlusion Percutaneous Revascularization. Cardiovasc Revasc Med. 2021; 33:32–40.

Intravascular imaging is essential for stent size selection, deployment, and optimization. IVUS and OCT can accurately measure the lumen diameter as well as the length of the vessel segment that should be covered with stents (Figures 9, 10). Stent under-sizing is among the strongest predictors of in-stent restenosis and thrombosis, whereas balloon or stent over-sizing predisposes to vessel perforation and distal edge dissections (50, 51). The selection of stent diameter is based on the measurement of the distal vessel diameter using a relatively healthy segment. Appropriate stent length is likewise important as failure to fully cover the diseased segment (geographical miss) has been associated with worse outcomes (52). Overtreatment of the distal segment with the implantation of multiple stents should also be avoided (2). Studies based on IVUS imaging have shown significant increase in lumen size distal and positive remodeling after successful CTO recanalization (3, 53). Positive vessel remodeling is caused mainly because of the enlargement of the external elastic membrane and at a lesser degree due to plaque burden reduction (53). Intravascular imaging can help to determine whether the vessel distal to the CTO is severely diseased and thus treated or has negative remodeling and will likely increase in size during follow up (54). Defer stenting in the later scenario is the best option as implanting a stent that will likely prove to be undersized at follow-up will increase the likelihood of stent failure. Intravascular imaging modalities can recognize the formation of compressive hematomas in the vessel wall distal to an implanted stent. In this case, cutting balloons can be used for haematoma decompression, whilst stent placement should be avoided to prevent further distal propagation of the subintimal haematoma and subsequent poor distal run off (29). Gomez-Lara et al. propose a conservative approach, based on intensive statin therapy, for non-flow-limiting lesions located distal to a successfully recanalized CTO reserving PCI with low dilatation pressure for flow-limiting stenoses (53). In such cases, scheduled angiographic follow-up will determine the need for further stenting.

Post-stenting, intravascular imaging can reveal areas of stent underexpansion, malapposition, dissection or incomplete lesion coverage that will likely lead to adverse events if they are left untreated. Stent underexpansion and malapposition are treated with post-dilatations with non-compliant balloons, whilst dissection or incomplete lesion coverage require further stenting.

Two randomized controlled clinical trials have shown improved outcomes with IVUS-guided over angiographic-guided PCI in the setting of CTO PCI. The Korean Chronic Total Occlusion Intervention with Drug-eluting Stents guided by IVUS (**CTO-IVUS**) randomized 402 patients after successful guidewire crossing of the CTO to receive IVUS guided PCI vs. angiographic-guided PCI. Although cardiac mortality was similar, the use of IVUS reduced major adverse cardiovascular events (MACE) at 12 months (2.6% vs. 7.1%; *p* = 0.035) (55). Likewise, The Comparison of Angiography—vs. IVUS—guided Stent Implantation for Chronic Total Coronary Occlusion Recanalization **(AIR-CTO**) randomized 230 patients after successful guidewire crossing to IVUS—vs. angiography—guided PCI, reporting lower rates of 12-month in-stent late lumen loss (0.28 ± 0.48 mm vs. 0.46 ± 0.68 mm, *p* = 0.025) and lower rates of stent restenosis (3.9% vs.13.7%, *p* = 0.021) in the IVUS-guided group in patients with true to true wire crossing and true intraplaque stent implantation. Although the study was not powered for clinical outcomes, IVUS utilization led to a lower rate of definite/probable stent thrombosis at 2 years (0.9% vs. 6.1%, *p* = 0.043) (56).

A recent metanalysis that included the two previously mentioned RCTs and two observational studies (total population: 1975 patients) showed that IVUS-guided CTO PCI was associated with a lower risk of stent thrombosis (odds ratio, 0.24; 95% confidence interval, 0.08–0.76; *p* = 0.02; *I*^2^ = 0%), shorter procedural time (*p* < 0.001; *I*^2^ = 88%), shorter fluoroscopy time (*p* < 0.001; *I*^2^ = 63%), and less contrast volume use (*p* < 0.001; *I*^2^ = 59%). Furthermore, IVUS utilization led to implantation of fewer stents and shorter total stent length. Nevertheless, all-cause mortality, cardiovascular mortality, MACE, myocardial infarction, and target vessel revascularization were similar between the two groups (57). The major studies regarding the use of IVUS for stent implantation/optimization in CTO PCI are outlined in Table 2.

**Table 2:** Major studies examining the usefulness of intravascular imaging in chronic total occlusion interventions.

Study	Publication year	Study type	Number of patients	Use of IVUS	MACE	Major findings
Kim et al. (IVUS-CTO)	2015	RCT	402 (IVUS rate 50%)	Guiding PCI after successful crossing	Composite of cardiac death, MI, TVR	At 12-month follow-up MACE rates were significantly lower in the IVUS-guided arm
Tian et al. (AIR-CTO)	2015	RCT	130 (IVUS rate 50%)	Penetration to true lumen + stent optimization	Not reported	12-month in-stent LLL and in true lumen restenosis were lower in the IVUS-gated arm
Hong et al. (From the multicenter K-CTO)	2014	Observational	402 (IVUS rate 50% in the propensity score-matched population and 39% in the overall population)	IVUS guidance pre- or/and post-stenting	According to ARC (including death, MI, ST, TLR)	At two years, MACE rates were similar between the two groups. IVUS-guided CTO PCI was associated with less ST and a trend towards less MI
Vemmou et al. (From the PROGRESS-CTO)	2020	Observational	941 (IVUS rate 37%)	Stent optimization	Composite of cardiac death, ACS and TVR	At 12 months the incidence of MACE was similar between the two groups, although the lesions were more complex in the IVUS-guided group
Kalogeropoulos et al. (Single center)	2021	Observational	514 (IVUS rate 36%)	Stent planning ± optimization	composite of all-cause death, cardiac death, MI and TVR	At up to 8 years follow-up, MACE was similar between the two groups. IVUS led to the implantation of stents with larger diameter and longer stented segments

ARC, Academic Research Consortium; IVUS, intravascular ultrasound; LLL, late lumen loss; MACE, major adverse cardiovascular events; MI, myocardial infarction; PCI, percutaneous coronary intervention, RCT, randomized controlled trials; ST, stent thrombosis; TLR, target lesion revascularization; TVR, target vessel revascularization.

### 1.5. The role of OCT

Due to the previously described inherent limitations, OCT has been investigated less extensively compared to IVUS, with respect to CTO interventions. Inability for real time imaging excludes the use of OCT for re-entry facilitation in the setting of proximal cap ambiguity or difficult reverse CART scenarios. In contrast, given its high-resolution, OCT is an ideal tool for detecting thrombus, tissue protrusion, stent malapposition and edge dissections after successful stent placement (9). Therefore, the role of OCT is limited as an investigational tool to assess intravascular healing post CTO-PCI rather than in every day clinical practice, where IVUS is mostly used. OCT imaging has revealed high rates of stent strut malapposition and incomplete stent strut coverage after CTO PCI (58). In the ALSTER CTO-OCT registry (Delayed DES endothelialization after subintimal recanalization of chronic of total occlusion: Observation by optical coherence tomography) patients who had undergone PCI for CTO lesions were shown to have higher rates of strut malapposition and delayed stent strut coverage vs. patients with non-CTO lesions at clinically driven angiographic follow-up (6.5 ± 2.1 months post PCI) (59). Similar results were shown in another study, conducted by Jia et al, where routine OCT was performed in the index procedure, as well as 6 months later: malapposed struts, tissue protrusion and intra-stent thrombus rates were higher after CTO PCI vs. non-CTO PCI. In addition, at 6-months follow-up, malapposition and cross sections with uncovered struts were most frequently observed in the CTO group (60). In the CONSISTENT study, the quality of intravascular healing after successful CTO PCI, as assessed by strut coverage on OCT at 12 months, was comparable between patients in whom dissection and re-entry techniques were utilized and subintimal stenting was performed and those in whom true intraplaque stent implantation was performed, highlighting that subintimal stenting does not predispose to stent failure after CTO PCI and intravascular imaging has a potentially more determinant role in ameliorating long-term outcomes after successful CTO PCI (48).

## 2. Conclusion

IVUS is the preferred imaging modality for CTO PCI given the inherent limitations of OCT. Intravascular imaging can facilitate CTO crossing being especially useful in the most complex subsets where conventional angiography cannot effectively guide the advancement of the guidewire through the occlusion. Stent optimization with the use of IVUS has improved clinical outcomes and is strongly recommended in essentially every CTO PCI. Emphasis should be given on interventionalists' training since unfamiliarity with the IVUS techniques that are used for crossing facilitation and image interpretation are among the main barriers for intravascular imaging usage.
